# Migrating Polyarthritis as a Feature of Occult Malignancy: 2 Case Reports and a Review of the Literature

**DOI:** 10.1155/2015/934039

**Published:** 2015-10-19

**Authors:** Geoffrey Alan Watson, Lorraine O'Neill, Ruth Law, Geraldine McCarthy, Douglas Veale

**Affiliations:** ^1^St. Vincent's University Hospital, Dublin, Ireland; ^2^Mater Misericordiae University Hospital, Dublin, Ireland

## Abstract

Malignant disease may be associated with a wide variety of musculoskeletal syndromes. Rarely the musculoskeletal system can be indirectly affected by paraneoplastic phenomena, such as carcinomatous polyarthritis (CP). The differential diagnosis for CP is broad and is often a diagnosis of exclusion. CP often presents similarly to other forms of inflammatory arthritis, and a detailed history and physical examination can often distinguish CP from other more common causes of polyarticular arthritis. However serological tests such as rheumatoid factor (RF) and anti-citrullinated peptide (anti-CCP) antibody positivity, while rare, can be misleading. Clinical awareness and suspicion are paramount in achieving an accurate diagnosis and early detection of an occult neoplasm is critical for prompt management and therapy. We report two cases presenting with this unique clinical phenotype associated with paraneoplastic polyarthropathy and review the literature.

## 1. Introduction

Malignant disease may be associated with a wide variety of musculoskeletal syndromes [1–3]. Mechanisms include direct local invasion of the primary tumor, metastasis, and synovial response to juxta-articular masses. Rarely the musculoskeletal system can in addition be indirectly affected by paraneoplastic phenomena, such as carcinomatous polyarthritis (CP). CP is a poorly understood clinical entity and may present similarly to other forms of inflammatory arthritis. Distinguishing features have been proposed and include late age and abrupt onset, asymmetric joint involvement, negative family history, and resolution of symptoms after diagnosis and treatment of the underlying malignancy [2]. However CP remains a diagnosis of exclusion and positive serological tests such as rheumatoid factor (RF) may be misleading. It has previously been postulated that the absence of anti-citrullinated peptide (anti-CCP) antibodies may distinguish CP from other more common rheumatic conditions such as rheumatoid arthritis (RA) [2]. However, while rare, isolated case reports are emerging linking CP with anti-CCP positivity, suggesting that serology may not be wholly reliable in providing this distinction. Here we report two cases presenting with a polyarthropathy including one case where anti-CCP antibodies were detectable.

## 2. Case Reports

Our first case involves 80-year-old female who presented with a three-day history of right-sided shoulder pain and associated fever. On further questioning the patient described asymmetric migratory joint pain over the preceding three-week period, affecting her left wrist, knees, and right shoulder. She had been taking nonsteroidal anti-inflammatory drugs (NSAIDs) without relief and was also receiving a two-week course of ciprofloxacin for a urinary tract infection. Physical examination revealed tenderness and painful restriction of motion of the right shoulder. There was also a right-sided knee effusion. The remainder of the physical examination was unremarkable.

Her medical history was significant for type 2 diabetes, ischaemic heart disease, and congestive heart failure. She was a lifelong nonsmoker and did not consume any alcohol. Review of systems was otherwise unremarkable.

Initial laboratory results revealed a white cell count of 11.9 × 10^9^/L, hemoglobin 11.2 g/dL, and platelets 384 × 10^9^/L. A metabolic panel was significant for mild hyponatremia (131 mmol/L) (normal levels 135–145 mmol/L) and a slightly raised urea (7.7 mmol/L) and creatinine (93 *μ*mol/L). Her erythrocyte sedimentation rate (ESR) was 21 mm/h and C-reactive protein (CRP) was elevated at 111.8 mg/L. Uric acid was 0.29 mmol/L. Rheumatoid factor was marginally elevated at 42 IU/mL. Anti-CCP was also elevated at 36 IU/mL. An X-ray of her right shoulder showed moderate degenerative changes at the acromioclavicular joint. Imaging of the knees and pelvis showed mild degenerative changes. Urine dipstick was positive for leukocytes but negative for nitrites. She was initially suspected of having polyarticular gout based on her slightly elevated uric acid levels and was subsequently treated with colchicine.

**Figure 1:**
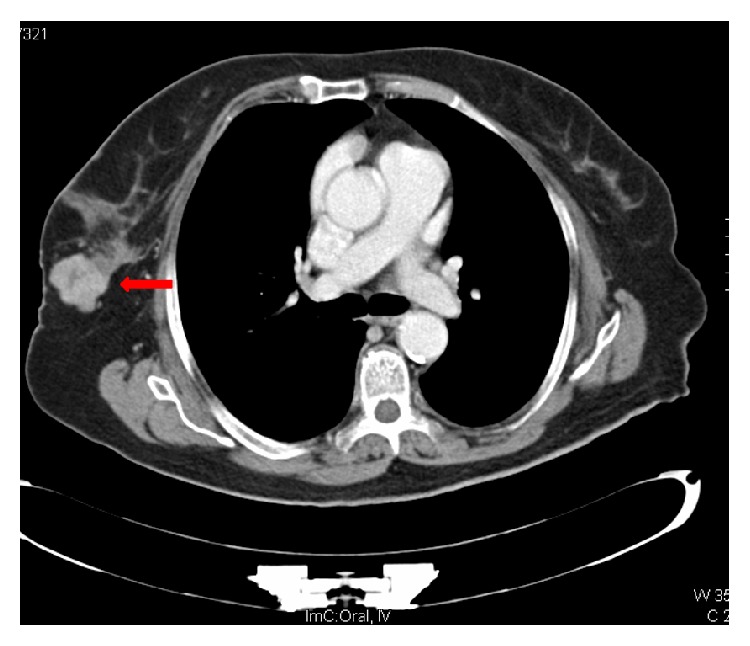
CT of abdomen. The red arrow illustrates a well circumscribed, multilobulated 3.3 cm lesion in the inferior outer quadrant of the right breast.

**Figure 2:**
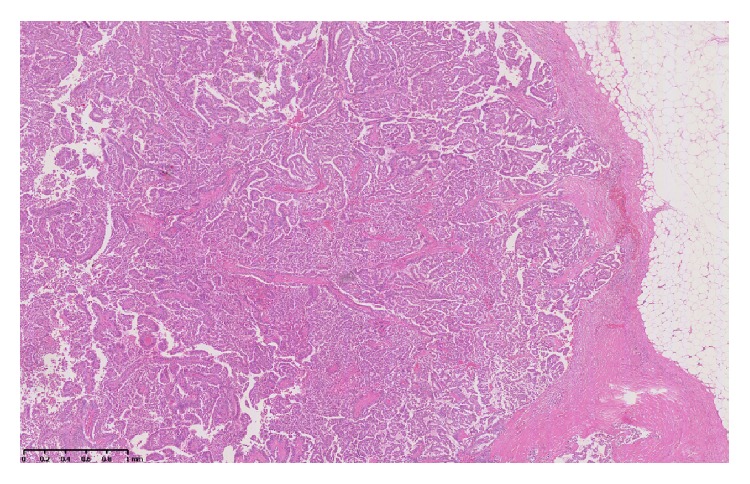
Encapsulated papillary carcinoma showing classical papillary architecture with surrounding fibrous capsule.

During her admission she developed nonspecific abdominal pain and tenderness in the right iliac fossa. A CT of abdomen was requested and reported no intra-abdominal pathology. However there was an incidental finding of a 3.3 cm multilobulated lesion in the inferior outer quadrant of the right breast (Figure 1). Subsequent mammogram revealed a 3.5 cm irregular hypoechoic mass highly suggestive of malignancy. This was confirmed after tissue biopsy, with microscopy revealing a papillary carcinoma (Figure 2).

During that time her right shoulder pain spontaneously resolved. However she then developed new onset right wrist pain, swelling, and erythema. X-ray revealed moderate degenerative changes. Joint aspiration revealed markedly increased inflammatory cells but no crystals. Colchicine and NSAIDs were stopped and she was given IM methylprednisolone to which she responded well, and her CRP decreased to 70. She was seen in clinic one week later and had no appreciable synovitis on examination.

Six weeks later she underwent chemotherapy and radiotherapy for her breast cancer. She was seen again in clinic ten weeks later and she did not report any recurrence of her joint symptoms.

Our second patient, 71-year-old female, was referred by her general practitioner for evaluation of a six-month history of pain and swelling of her hands and feet. This was associated with early morning stiffness lasting greater than two hours daily. NSAIDs and a short course of prednisolone had failed to improve her symptoms.

Her past medical history was significant for ischaemic heart disease, hypertension, hyperlipidaemia, and peptic ulcer disease. She had a thirty-pack year history of smoking. Three of her four siblings had been treated for colorectal or breast neoplasms.

On examination she had thirteen tender and ten swollen joints including metacarpophalangeal (MCP), proximal interphalangeal (PIP), wrist, shoulder, ankle, and knee joints. Grade 2 clubbing was evident as was a multinodular goitre. Other than a raised CRP at 12 mg/L and serum calcium at 2.68 mmol/L, baseline laboratory investigations were normal. Rheumatoid factor and anti-CCP antibodies were both negative. Chest radiograph revealed a left upper lobe lesion, which was confirmed on CT. Subsequent biopsy identified a non-small cell adenocarcinoma. She underwent a lobectomy with a rapid resolution of her joint symptoms.

## 3. Discussion

Migratory polyarthritis is a common presentation with a broad differential diagnosis [1–4]. Common causes include inflammatory arthritis including crystal arthropathy and other forms of systemic rheumatic disease such as connective tissue disorders and occasionally infectious agents [4]. Less commonly, joint symptoms may be attributed to neoplastic disease.

CP is associated with a wide variety of solid tumours and haematologic malignancies, particularly cancers of the lung and ovaries (Table 1). Cancer polyarthritis was first described by Lansbury in 1953 [5] and by MacKenzie and Scherbel in 1963 [6]. The topic has since been reviewed by Naschitz et al. [7] and most recently by Larson et al. [8]. Hypertrophic osteoarthropathy, dermatomyositis, paraneoplastic vasculitis, and most recently palmar fasciitis have all been described as paraneoplastic disorders [1, 9]. However, carcinomatous polyarthritis remains a rare and poorly understood clinical entity [2, 3]. CP is historically defined by the following criteria [1, 2]:

- It must occur during the course of an identified malignant disease or precede clinical evidence of a malignancy.
- Symptoms cannot be the result of direct tumor invasion or compression.
- Symptoms improve with treatment of the underlining neoplasm.

In 1985, Caldwell and McCallum published the key features of cancer polyarthritis and suggested distinguishing features to aid in narrowing the differential [57]. Pfitzenmeyer et al. [58] later added the absence of characteristic radiologic lesions to the list of typical features (Box 1). These features have been supported by recent case reviews [2, 3, 8, 10]. It is now well recognised that rheumatic syndromes often precede the diagnosis of malignant disease [1–3, 8, 57]. Racanelli et al. found that, in patients with solid tumours, only 11.5% had a malignancy diagnosed at the time of their rheumatic presentation, while 88.5% were diagnosed after their initial presentation [59]. Thus new presentations of polyarthropathy may represent an important clue in the detection of an occult malignancy.

CP however remains a diagnosis of exclusion and must be distinguished from other more common causes of polyarthritis. Infectious causes due to bacteria or Lyme disease may be associated with fever, acute symptom onset, and monoarthritis. A thorough clinical history is useful in detecting seronegative spondyloarthropathies. Reactive arthritis is usually preceded by an infection such as campylobacter-induced diarrhea, while enteric arthritis may be seen in patients with a history of inflammatory bowel disease. Finally the crystal arthropathies can be easily distinguished by joint aspiration and visualisation of crystals under the microscope.

Distinguishing CP from a late onset form of RA, however, is challenging. Previous studies have shown that the incidence of both increases with age. Both may present over the course of weeks to months with similar signs and symptoms, namely, soft tissue swelling, limited range of motion of affected joints, and morning stiffness [3, 9, 11–16]. Features suggestive of chronic disease and inflammation (anaemia, elevated ESR/CRP) are also seen [2, 3, 11–13, 16, 20, 22, 28, 29, 42, 49, 55]. The joint fluid generally shows nonspecific inflammatory changes [1, 2, 11, 16, 17]. Imaging studies are largely unremarkable [1–3, 14, 16, 60]. In addition symptoms often fail to resolve with the use of conventional therapy such as NSAIDs and steroids [2, 27–32, 41, 42, 44, 48, 50].

Several case reports included in Box 1 have emerged documenting findings that challenge the traditional distinguishing features of CP. Several noted symmetric joint involvement involving the wrists, knees, and small joints of the hands as the predominant rheumatic manifestation [2, 3, 8–16, 23, 27, 28, 31, 34, 35, 38–40, 42, 43, 48, 49].

Our first case had a positive rheumatoid factor, which can be partially explained by the underlying malignancy, which is associated with a positive rheumatoid factor in 10–20 percent of patients [58]. Morel et al. documented symptom relief for 9 out of 20 patients who were treated with NSAIDs, while most responded to steroid therapy [60].

An additional means to differentiate these two disorders was postulated by measuring anti-cyclic citrullinated peptide (anti CCP) antibodies. Anti-CCP has a similar sensitivity to RF (50–75%) with a higher specificity (90–95%) [61]. Due to this high specificity, however, a positive Anti-CCP may result in an inaccurate diagnosis of rheumatoid arthritis, thus resulting in delayed diagnosis and treatment of the underlying malignancy. To our knowledge, only four case reports, including the present case, have reported an association between CP and elevated anti-CCP levels [8, 18, 19] (Box 1). Thus, many features that have been proposed to distinguish between CP and RA are not always reliable.

One prevailing theme however is resolution of rheumatic symptoms after treatment of the underlying malignancy [2, 3, 9, 11–16, 22, 27, 28, 33–35, 39–41, 43–48, 50, 52, 55]. Resolution of symptoms after chemotherapy or surgery may vary, and while our first patient's symptoms may have been masked by steroids, our second patient showed a rapid improvement just days after undergoing surgery. The return of arthritic symptoms in these patients should provoke further investigation to rule out any evidence of tumour recurrence [2].

To date, the pathogenesis of this disorder remains unclear. Bradley and Pinals suggested that circulating immune complexes (CIC), which have been observed in over 60 percent of some types of cancers, may play a pivotal role [11]. Theories suggesting that elevated levels of platelet activating factors may be deposited in the synovium and trigger an inflammatory response have also been postulated [2, 15]. Additional mechanisms have been proposed including autoimmune phenomena involving lymphocytes originating in hyperplastic lymph nodes draining tumour sites [1, 9, 11, 14]; however these theories all remain speculative.

## 4. Summary

Carcinomatous polyarthritis is a rare disorder associated with a wide variety of malignancies. The differential diagnosis for CP is broad and is often a diagnosis of exclusion. Several of the classical features initially proposed to distinguish CP may be unreliable, and positive serological tests such as rheumatoid factor and anti-CCP antibody positivity can be misleading. A comprehensive history and physical examination is paramount in distinguishing CP from other more common causes of polyarticular arthritis, with careful attention to social and family history to detect any possible risk factors for cancer. Clinical awareness and suspicion remain the most important factors in achieving an accurate diagnosis. Early diagnosis of an occult neoplasm is critical for prompt management and therapy and can ultimately be lifesaving.

## Figures and Tables

**Box 1:**
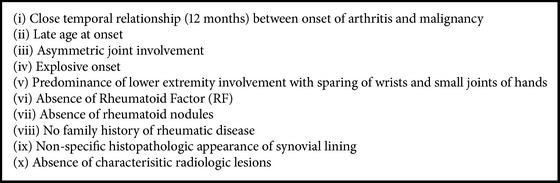
**Box 1: **Features of cancer polyarthritis (Stummvoll et al. [3], Larson et al. [8], Caldwell and McCallum [57], and Pfitzenmeyer et al. [58]).

**Table 1:** Malignancies associated with carcinomatous polyarthritis. Four cases, highlighted in bold, have shown anti-CCP positivity. Rheumatoid factor (RF), anti-citrullinated protein antibody (anti-CCP), positive (+), negative (−), not included (NI), Non-Small Cell Lung Cancer (NSCLC), Squamous Cell Cancer (SCC), Small Cell Lung Cancer (SCLC), Acute Lymphoblastic Leukemia (ALL), Chronic Myeloid Leukemia (CML), and Chronic Lymphoid Leukemia (CLL).

Case report and reference	Type of malignancy	RF	Anti-CCP
**Present case **	**Breast**	**+**	**+**
Present case	NSCLC	−	−
Zupancic et al. [2]	SCC lung	+	NI
Stummvoll et al. [3]	Adenocarcinoma colon	−	NI
Stummvoll et al. [3]	SCC Lung	−	NI
**Larson et al. [8]**	**NSCLC (adenocarcinoma)**	**+**	**+**
Nadal et al. [10]	Prostate cancer	NI	−
Bradley and Pinals [11]	Spindle cell cancer	+	NI
Pines et al. [12]	Breast	−	NI
Pines et al. [12]	Unknown primary	−	NI
Pines et al. [12]	SCLC	−	NI
Acosta Madiedo et al. [13]	NSCLC	−	NI
Chuan et al. [9]	Tubular adenocarcinoma stomach	−	NI
Eggelmeijer and Macfarlane [14]	Supraglottic SCC	−	NI
Bennett et al. [15]	Ovarian adenocarcinoma	+	NI
Simon and Ford [16]	Adenocarcinoma colon	−	NI
Mok and Kwan [17]	Unknown primary	+	NI
**Handy et al. [18]**	**T cell ALL**	**+**	**+**
**Kumar et al. [19]**	**Pancreas **	**+**	**+**
Sheehy et al. [20]	NSCLC (adenocarcinoma)	+	NI
Bivalacqua et al. [21]	NSCLC (adenocarcinoma)	NI	NI
Docquier et al. [22]	Uterine adenocarcinoma	−	NI
Haroon and Phelan [23]	Pancreatic cancer	−	NI
Ardalan and Shoja [24]	Multiple myeloma	−	NI
Leslie [25]	Cervical cancer	NI	NI
Baijens and Manni [26]	cT4N2cM0 hypopharynx carcinoma	NI	NI
Martorell et al. [27]	Serocystadenocarcinoma ovary	−	NI
Martorell et al. [27]	Serocystadenocarcinoma ovary	+	NI
Martorell et al. [27]	Ovarian carcinoma	−	NI
Martorell et al. [27]	Serocystadenocarcinoma ovary	−	NI
Medsger et al. [28]	Ovarian adenocarcinoma	NI	NI
Medsger et al. [28]	Ovarian adenocarcinoma	NI	NI
Medsger et al. [28]	Ovarian adenocarcinoma	NI	NI
Medsger et al. [28]	Ovarian adenocarcinoma	NI	NI
Medsger et al. [28]	Ovarian adenocarcinoma	NI	NI
Medsger et al. [28]	Ovarian adenocarcinoma	NI	NI
Baron [29]	SCLC	−	NI
Baer and Phillips [30]	Pancreatic adenocarcinoma	NI	NI
Taggart et al. [31]	Adenocarcinoma Fallopian tube	−	NI
Taggart et al. [31]	Ovarian adenocarcinoma	NI	NI
Michaels and Sorber [32]	Pancreatic adenocarcinoma	−	NI
Shiel et al. [33]	SCLC	NI	NI
Shiel et al. [33]	Ovarian adenocarcinoma	NI	NI
Pfinsgraff et al. [34]	CML	−	NI
Pfinsgraff et al. [34]	Pancreatic adenocarcinoma, parathyroid adenoma	NI	NI
Pfinsgraff et al. [34]	Squamous cell carcinoma unknown primary	−	NI
Pfinsgraff et al. [34]	Adenocarcinoma unknown primary	NI	NI
Pfinsgraff et al. [34]	Hodgkin disease	NI	NI
Valverde-Garcia et al. [35]	Breast cancer	NI	NI
Cammilleri et al. [36]	Follicular B cell lymphoma	−	NI
Willemse et al. [37]	Adenocarcinoma of the coelomic epithelium	−	NI
Van den Bergh et al. [38]	Adenocarcinoma of prostate prolactinoma	−	NI
Mathieu et al. [39]	CLL	NI	NI
Vinker et al. [40]	Ovarian adenocarcinoma	NI	NI
Saxman and Seitz [41]	Breast carcinoma	−	NI
Grados et al. [42]	Transitional cell carcinoma of the renal pelvis	−	NI
Grados et al. [42]	Adenocarcinoma of the uterus	−	NI
Enomoto et al. [43]	Early-stage gastric carcinoma	+	NI
Denschlag et al. [44]	Fallopian tube carcinoma	−	NI
Giannakopoulos et al. [45]	Ovarian adenocarcinoma	−	NI
Yogarajah et al. [46]	Ovarian adenocarcinoma	NI	NI
Bolibar et al. [47]	Ovarian adenocarcinoma	−	NI
Preda et al. [48]	Ovarian adenocarcinoma	NI	NI
Krishna et al. [49]	Breast cancer	−	NI
Qureshi and Saavedra [50]	Ovarian adenocarcinoma	−	NI
Clarke et al. [51]	Transitional cell carcinoma of the bladder	−	NI
Nahar and Al-Rajhi [52]	Ovarian adenocarcinoma	−	NI
Sandhya and Danda [53]	Breast carcinoma	NI	NI
Mcgivern and Mcaleese [54]	NSCLC	NI	NI
Salmon et al. [55]	Ovarian adenocarcinoma	−	NI
Salmon et al. [55]	Ovarian adenocarcinoma	−	NI
Salmon et al. [55]	Uterine cancer	NI	NI
Shetty et al. [56]	Neuroendocrine tumour of adrenal gland	−	NI
